# Cancer Core Europe: A translational research infrastructure for a European mission on cancer

**DOI:** 10.1002/1878-0261.12447

**Published:** 2019-02-02

**Authors:** Alexander M. M. Eggermont, Giovanni Apolone, Michael Baumann, Carlos Caldas, Julio E. Celis, Francesco de Lorenzo, Ingemar Ernberg, Ulrik Ringborg, John Rowell, Josep Tabernero, Emile Voest, Fabien Calvo

**Affiliations:** ^1^ Gustave Roussy Cancer Campus Grand Paris Villejuif, France Cancer Core Europe France; ^2^ Fondazione IRCCS Istituto Nazionale dei Tumori di Milano Milan Italy; ^3^ German Cancer Research Center (DKFZ) Heidelberg Germany; ^4^ Department of Oncology Cancer Research UK Cambridge Institute University of Cambridge UK; ^5^ Danish Cancer Society Research Centre Copenhagen Denmark; ^6^ European Cancer Patient Coalition Brussels Belgium; ^7^ Italian Federation of Cancer Patients Organisations Rome Italy; ^8^ Cancer Centre Karolinska Karolinska Institutet Stockholm Sweden; ^9^ Institute of Oncology (VHIO) Vall d'Hebron University Hospital Barcelona Spain; ^10^ Netherlands Cancer Institute Amsterdam The Netherlands; ^11^ Cancer Core Europe Villejuif France

**Keywords:** alliance, cancer research, infrastructure, innovation, oncopolicy

## Abstract

Cancer Core Europe is a European legal alliance consisting of seven leading cancer centres – most of them Comprehensive Cancer Centres (CCCs) – with a single portal system to engage in various research projects with partners. Cancer Core Europe was established to create a sustainable, high‐level, shared research infrastructure platform hosting research collaborations and task forces (data sharing, clinical trials, genomics, immunotherapy, imaging, education and training, and legal and ethical issues), with a controlled expansion agenda. Translational cancer research covers the cancer research continuum from basic to preclinical to early clinical, late clinical, and outcomes research. Basic–preclinical research serves as the ‘engine’ for early clinical research by bridging the early translational research gap and is the primary and current focus of the consortium as exemplified by the launching of the Basket of Baskets trial, Europe's largest precision cancer medicine trial. Inspired by the creation of Cancer Core Europe, the prevention community established Cancer Prevention Europe, a consortium of ten cancer prevention centres aimed at supporting the complete prevention research continuum. Presently, Cancer Core Europe and Cancer Prevention Europe are integrating therapeutics and prevention strategies to address in partnership the widening cancer problem. By providing innovative approaches for cancer research, links to healthcare systems, development of quality‐assured multidisciplinary cancer care, and assessment of long‐term outcomes, the virtual infrastructure will serve as a hub to connect and interact with other centres across Europe and beyond. Together, Cancer Core Europe and Cancer Prevention Europe are prepared to function as a central engine to tackle, in collaboration with various partners, a potential ‘mission on cancer’ addressing the cancer burden.

**Abbreviations**
BOB: Basket of Baskets trial
CCC: comprehensive Cancer Centre
CPE: cancer prevention Europe
ctDNA: circulating tumour DNA
CTscan: computerized tomography scan
DKTK: German Cancer Consortium
ECPC: European Cancer Patient Coalition
EU: European Union
FP6: Framework Program 6
FP7: Framework Program 7
PET: positron emission tomography
RNAseq: RNA sequencing
SIRIC: Sites de recherche intégrée sur le cancer

## 1 An alarming increase of the cancer problem in Europe and the World

Europe is facing a significant burden in connection with cancers. While representing less than 10% of the global population, our continent is dealing with a quarter of all cancers. Moreover, these numbers are expected to increase due to our ageing societies. It is estimated that over 3.9 million Europeans will be diagnosed with cancers in 2018 and that almost 2 million in the same year will die from these diseases. A majority of cancer patients (55%) will gain a prolonged disease‐free survival, and cure, but big killers with a relatively low impact of treatment remain, such as lung cancer (20% of all cancer deaths), colorectal cancer, advanced breast cancer, and pancreatic cancer (Ferlay *et al*., 2018). These numbers are hiding important differences, observable in terms of incidence, stage at diagnosis, treatment opportunities, access to innovation of systemic treatments, and healthcare coverage, both at the country level (a divide between northern and western Europe and southern, central and eastern Europe) and at the regional level within a given country. Though the mortality rates decrease in the occidental part, they remain high in the more oriental regions of Europe. Similar data show that low‐income countries in the world are facing what looks like epidemics of cancers caused by the epidemiological transition (Corbett *et al*., 2018). In Europe, a 10% (around 20% according to the CPE manifest) increase in cancer incidence is expected in the next 15 years. As many as 13 million patients will be considered long‐term survivors in 2035, in need of professional and social support and demanding continuous research efforts in order to reduce long‐term side effects of treatments, to avoid secondary cancers and to facilitate full reintegration into society in all aspects.

## 2 The increasing economic burden of cancer

The indirect cost of cancer due to premature death and disability (i.e. excluding the medical expenses) is growing worldwide, being estimated at almost 900 billion USD in 2008 (Sullivan *et al*., 2011). However, no clear relationship between these high costs and the increasing incidence and mortality data has been established. In the EU countries, the economic burden was calculated at €126 billion in 2009, €52 billion in productivity loss, €37 billion on health care and €13 billion on drug expenses. Spending on cancer drugs has increased from €7.6 billion in 2005 to €19.1 billion in 2014 due to an increased number of patients treated better, but at very high costs (Jönsson *et al*., 2016; Luengo‐Fernandez *et al*., 2013). Moreover, there is a huge variation in the availability of drugs within different EU countries as well as a significant variation in their costs (Cherny *et al*., 2016). Comprehensive approaches to the various cancer diseases and their related expenses are therefore required in all fields, from cancer prevention to precision care, from basic research to clinical trials and cohort surveys, including the support from mathematics, informatics and social sciences. The patient contribution to research, treatment decisions and healthcare policies, individually and collectively through their representatives, is also mandatory to change the vision of cancer prevention and cure.

## 3 A cancer research continuum

Traditionally, progression from basic research to patient's treatment with new therapeutics is a long and fragmented road, with ad hoc bridges being required between each separated step. The process is not efficient regarding energy, interaction between research and the health care, patient involvement, and time or money, and a result, a universal paradigm shift is needed to challenge cancer effectively. Cancer research must develop new therapeutic approaches and also design fast, reactive and efficient clinical trials that spare the patients’ contributions, and the only way to do this efficiently is by streamlining the process. Creating a continuum that covers basic, preclinical, early and late clinical and outcomes research depends on having a common agenda and a willingness to share quality‐controlled data. Information and data generated in every phase must be accessible to speed up the knowledge process and prevent stalling. This concept is fulfilled by collaborations between CCCs, patient‐focused entities capable of undertaking every stage of the process.

## 4 Creating research infrastructures in Europe over time

There is a need to consolidate and focus the fragmented European research towards innovation within the fields of prevention and therapeutics, in order to address the healthcare, social and economic challenges of cancer. In 2006, a pivotal FP6 project, EUROCAN+Plus, provided the first step in the process towards a larger, more consistent effort to fight cancer, and at its conclusion in 2008, several key findings and ‘next steps’ were proposed. A paradigm shift was deemed necessary to eliminate the segmented process of cancer research with little interaction between each step of the research continuum, and the development of CCCs, institutions that link research with the healthcare system, was emphasized. Furthermore, it was concluded that there was a need for creating a uniform platform for translational cancer research to bring together enough centres to generate the critical mass of patients, expertise and resource required to make a significant breakthrough in cancer care.

Building on these suggestions, the FP7 EurocanPlatform network of excellence was launched in 2011, emphasizing the need to stimulate multisite translational cancer research policies. A direct outcome of this project was the formation of Cancer Core Europe in 2014, with the sole objective of developing a sustainable infrastructure for the next era of translational cancer research. Cancer Core Europe is now a legal entity including seven European large cancer centres, most of which are CCCs.

Inspired by the creation of Cancer Core Europe, the leaders of 10 research institutes with prominent cancer prevention programmes in Europe (IARC in France, and institutes in the UK, DK, NL, Germany, Sweden and Italy) recently established Cancer Prevention Europe (CPE) (Forman *et al*., 2018). CPE is a consortium of multidisciplinary centres focused on reducing cancer mortality and morbidity through development of prevention policies and early diagnostic techniques (Forman *et al*., 2018, article in the same issue). A partnership between Cancer Core Europe and CPE would encompass every facet required to tackle the cancer challenge at an unprecedented level. It would structure a European hub able to support and drive innovation in every sector, linking healthcare systems together, and ensuring alignment of policies towards prevention and translational research.

## 5 The Cancer Core Europe initiative

**Figure 1:**
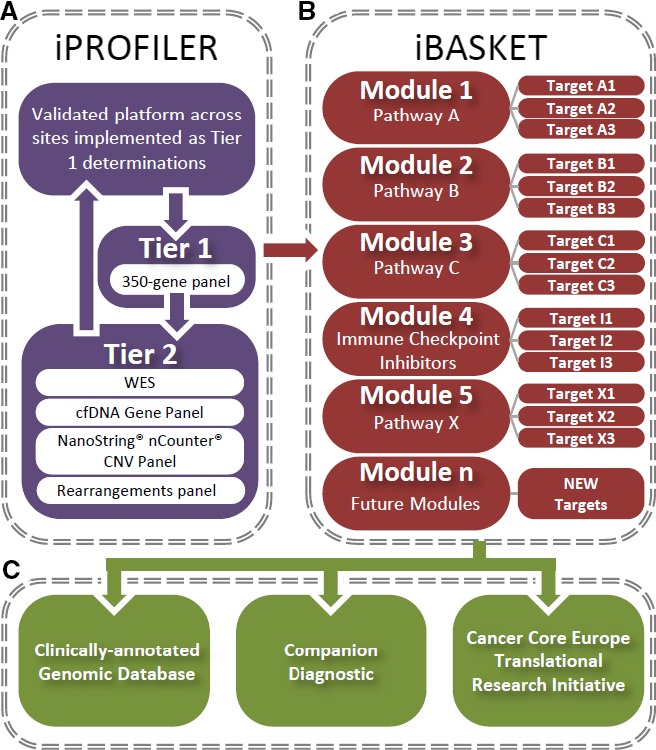
The Basket of Baskets Clinical Trial process. (A) iPROFILER: a 2‐tier process of increasing resolution to identify potential candidate and determine their molecular signature. (B) iBASKET: the array of modules in the trial process, each of which focuses on a different pathway. Within each module, there are several arms that target specific components of the modules pathway. (C) The outcomes of BOB.
- *Standardizing and developing radiomics*: The added value of modern functional and molecular imaging technologies and image analysis (radiomics) is another area of attention for the consortium; to analyse heterogeneity of metastatic disease and predict response to therapy, data must be shared following image harmonization between centres. Common clinical databases permit assessments of clinical effectiveness and outcomes on a longitudinal term based on health‐economics studies. Finally, educational activities are also considered to be essential: Cancer Core Europe is organizing an annual Summer School for Translational Cancer Research, offering a full‐week immersion of PhDs and MDs with experts from all over Europe to help them exchange ideas and projects. This school is open to all European students to train the next generation of leaders of translational and clinical oncology all over our continent and beyond. Further information is provided on the website: https://www.cancercoreeurope.eu/education-and-training/; see also article by I. Ernberg in this issue.
- *Patient participation:* The role of the patients themselves in the process is also of key importance since they are the start as well as the end point. The European Cancer Patient Coalition (ECPC) is working with the Cancer Core Europe Board of Directors to develop the best synergistic relationship with patients, with the objectives of information, complete awareness and participation in research projects, in treatment decision, follow‐up of care and outcomes, and therefore contributing to patient empowerment.
- *A legal entity with critical mass and single portal:* Cancer Core Europe is a consortium with a legal entity (alliance) which will support open science and sharing of resources. It has a rotating leadership; at present, Eggermont from Gustave Roussy is the chairman and Calvo the Chief Scientific Officer. The critical mass is substantial, with around 60 000 newly diagnosed patients, about 300 000 treated patients and approximately 1 200 000 consultations annually. Furthermore, over 1500 clinical trials are being conducted at the consortium. Cancer Core Europe will expand in the future, and the inclusion of new participants will be based on scientific excellence and the need for additional specific research geometries.
- *Designation of excellence programme:* The European Academy of Cancer Sciences has a quality assurance programme for translational cancer research designating CCCs of Excellence (Ringborg *et al*., 2018). In an ongoing process, the Cancer Core Europe participants use this process to quality‐assure translational research, and new participants are expected to use the same assessment procedure. In addition, each centre has the mission to work in its own country setting up collaboration with other CCCs.

- *Bottom‐up initiative*: Following the suggestion that a bottom‐up initiative would be required to make these changes at the European level, Alexander Eggermont (Gustave Roussy Cancer Campus Grand Paris) and Otmar Wiestler (DKFZ‐NCT, the German Center for Cancer Research and the National Center for Tumor Diseases, Heidelberg) spearheaded the creation of Cancer Core Europe in 2014 (Eggermont *et al*., 2014). By linking centres with robust basic/preclinical research with those having an outstanding experience in early phase clinical trials, it would be possible to set the agenda for addressing the translational research continuum, and to provide further care implementation and outcomes research focused on the innovative therapeutic needs required for balancing the growing cancer problem. The consortium now includes the Gustave Roussy Cancer Campus Grand Paris; Cambridge Cancer Centre, Cambridge; the Netherlands Cancer Institute, Amsterdam; Karolinska Institutet, Stockholm; the Vall d'Hebron Institute of Oncology, Barcelona; the German Center for Cancer Research and the National Center for Tumor Diseases, Heidelberg; and the Istituto Nazionale dei Tumori, Milano (Calvo *et al*., 2018).
- *Sharing infrastructures and sharing data*: Cancer Core Europe is a shared research infrastructure with research collaborations and task forces (common research data sharing expanded to the patient's care pathway observation and outcomes; innovative clinical trials associating large numbers of patients for fast results; shared genomic platform with common bioinformatics tools to provide usable molecular results and help medical tumour boards; imaging and radiomics; training and education, legal and ethical issues, and coordination across borders) that have representatives from all participating centres. The consortium is in the process of moving towards a virtual single ‘e‐hospital’ with shared translational research activities and compiled databases. Common clinical molecular profiling, initially developed in some centres, is now shared by all institutes and linked to a data centre, which enables computational biology to provide validated diagnostic information; in addition, standard operating procedures are currently under development for tissue and liquid biopsies along with ‘omics’ technologies, single‐cell analyses, functional genetics, immune profiling, proteomics, etc.
- *The basket of baskets trial (BOB):* Clinical and translational research with a focus on innovative precision cancer medicine handles a large number of specific subgroups of tumours characterized by individual molecular characteristics, and thus offers new possibilities to stratify patients for treatment. Given the large number of patient subgroups, traditional clinical trials methodology must move towards next‐generation clinical trials, and an integrated clinical trials structure is currently being used for a major precision cancer medicine initiative, known as ‘the Basket of Baskets trial’ (BOB; see Fig. 1). In brief, patients with refractory disease are screened for their genomic abnormalities using a 350‐gene panel on their initial tumour, followed up with ctDNA, and according to the results, directed to receive an appropriate treatment under different modules (defined by class of mutation‐driven signalling pathways, epigenetic modulations, hypermutated tumours, etc.) following the decision of the medical tumour board, with drugs being provided by pharma companies. When a recent biopsy is available, tumours are sequenced at multiple sites (whole‐genome sequencing and RNAseq in one platform) providing additional elements to the treatment decision, and discovery of new biomarkers. Inside a module addressing a particular gene pathway, there may be stratification according to different molecular alteration types. The biostatistics survey allows all these subgroups a limited number of inclusions which can be expanded if a response signal is observed. Additional modules can be implemented as amendments during the master protocol evolution, making it flexible and reactive. This protocol is, therefore, a long‐term, adaptable and expandable clinical trial, histology agnostic, allowing the development of biomarkers. All steps are under the expertise of a task force with quality control.

## 6 Collaboration and expansion for future perspectives in Europe and beyond

Collaboration between centres working at any level of the continuum is paramount for rapid advancement in tackling the challenges posed by cancer in all countries. The infrastructure created by Cancer Core Europe accelerates the research continuum and has designed a launching pad for advancing personalized and precision medicine. To further expand and share this infrastructure with all EU countries (and potentially beyond), Cancer Core Europe will grow by including additional centres at two levels. One central member will be identified per country to form the core network of Cancer Core Europe.

### 6.1 Expansion plan and initiation of associated members and networks

The expansion plan by Core Centres will be performed at a controlled rate because centres need time to prepare for membership with respect to structural, legal and collaborative requirements. The second expansion level is per country, identifying associated members for participation in translational research networks with the core institutes. Examples could be the DKTK network in Germany (see a separate chapter in this volume), the SIRIC centres network in France, the personalized cancer medicine network in the Netherlands, and the Comprehensive Cancer Centres network in Catalonia. Ultimately a rapidly increasing superstructure will emerge with supracritical mass to address outcomes research programmes in Europe and to create databases facilitating patient care and treatment through artificial intelligence‐driven discoveries (Lawler *et al*., 2018). Cancer Core Europe will collaborate with national and international research networks to increasingly cover all relevant research areas. CCCs will be pivotal for accelerating the development of potential clinical innovations. There must be at least one in every EU nation, and they must be more closely tied to the existing clinical research networks, improving the evaluation of the benefits for patients and the healthcare system in the later stages of the continuum.

### 6.2 Programmes to expend east and southeast wards

In order to accelerate construction of a bridge with centres in Eastern Europe, Cancer Core Europe members will each build one‐on‐one developmental programmes with designated centres to address their needs and to accompany them through bilateral education and training programmes and infrastructure building programmes to accelerate their development towards Cancer Core Europe membership.

### 6.3 Transfer of expertise

Cancer Core Europe also represents a vast wealth of transferable expertise. Any centre joining will have access to all Cancer Core Europe activities, which will, in turn, expand the individual centres’ competences. Centres currently without the scope to run trials will be able to do so through the clinical trials platform, and the close connection to all levels of the translational research continuum will benefit and expand on the potential of cancer research as a whole. In essence, a borderless translational research entity will be formed, providing the critical mass of expertise, patients and utilities, also allowing for the expansion of activities to include the advancement of diagnostics, prevention, healthcare policies, socio‐economic ramifications, patient rights and survivorship care.

## 7 A mission on cancer will provide new hope

In 2017, a mission on cancer was proposed, stating that ‘by combining innovative prevention and treatment strategies in a sustainable state‐of‐the‐art virtual European cancer centre/infrastructure, it will be possible by 2030 to achieve long‐term survival in three out of four cancer patients in countries with well‐developed healthcare systems. Furthermore, the concerted actions will pave the way to handling the economic and social inequalities in countries with less‐developed systems’ (Celis and Pavalkis, 2017). In June 2018, a press release from the European Commission concerning Horizon Europe stated that ‘Examples of missions could range from the fight against cancer, to clean transport or plastic‐free oceans. Missions will be codesigned with citizens, stakeholders, the European Parliament and the Member States’ (EU Press release, 2018). The initiation of a mission on cancer directed towards all aspects of cancer health care (the research continuum, prevention, health economics, social impact, patient well‐being and rights, infrastructure development such as CCCs, etc.) is the most viable option to instigate real change in the field and to demonstrate the social impact of research.

## 8 Future perspectives: How do we get there?

Clearly a lot of work needs to be done to have a successful integration of the different components of a mission on cancer. Such an integration will need collaborations with a variety of networks. The uniqueness of Cancer Core Europe makes it the ideal ‘engine’ for a mission on Cancer. The bottom‐up approach, the fusion of basic and preclinical research, will drive innovation forward, and the clinical trial platform offers an efficient process for rapid completion. The cooperation between Cancer Core Europe and Cancer Prevention Europe will be essential to expand the continuum to cover prevention, making it imperative also to involve patient advocacy groups like ECPC, and to work alongside policymakers at the national and European levels, whereby the longevity of the mission will be ensured, making it mandatory to elicit effective social change. Overall, a successful mission on cancer must benefit the advancement of cancer research and of care for all.

## Conflict of interest

In the past 5 years, Dr. Baumann attended an advisory board meeting of MERCK KGaA (Darmstadt), for which the University of Dresden received a travel grant. He further received funding for his research projects and for educational grants to the University of Dresden by Teutopharma GmbH (2011‐2015), IBA (2016), Bayer AG (2016‐2018), Merck KGaA (2016‐2030), Medipan GmbH (2014‐2018).

Dr. Baumann, as former chair of OncoRay (Dresden) and present CEO and Scientific Chair of the German Cancer Research Center (DKFZ, Heidelberg), signed/s contracts for his institute(s) and for the staff for research funding and collaborations with a multitude of companies worldwide.

For the German Cancer Research Center (DKFZ, Heidelberg) Dr. Baumann is on the supervisory boards of HI‐STEM gGmbH (Heidelberg).

For the present study, Dr. Baumann confirms that none of the above mentioned funding sources were involved in the study design or materials used, nor in the collection, analysis and interpretation of data nor in the writing of the paper.

Alexander M. M. Eggermont declares Honoraria over the last 5 years for any speaker, consultancy or advisory role from: Actelion, Agenus, Bayer, BMS, CellDex, Ellipses, Gilead, GSK, HalioDX, Incyte, IO Biotech, ISA pharmaceuticals, MedImmune, Merck GmbH, MSD, Nektar, Novartis, Pfizer, Polynoma, Regeneron, RiverDx, Sanofi, Sellas, SkylineDx

For the present study, Dr. Eggermont confirms that none of the above mentioned funding sources were involved in the study design or materials used, nor in the collection, analysis and interpretation of data nor in the writing of the paper.

## Author contributions

AMME, UR, and FC : Wrote first draft. Draft was sent out to all co‐authors and received comments from all co‐authors leading to final draft, that was approved by all co‐authors.
